# Video quality assessment using motion-compensated temporal filtering and manifold feature similarity

**DOI:** 10.1371/journal.pone.0175798

**Published:** 2017-04-26

**Authors:** Yang Song, Mei Yu, Gangyi Jiang, Feng Shao, Zongju Peng

**Affiliations:** Faculty of Electrical Engineering and Computer Science, Ningbo University, Ningbo, Zhejiang, China; Huazhong University of Science and Technology, CHINA

## Abstract

Well-performed Video quality assessment (VQA) method should be consistent with human visual systems for better prediction accuracy. In this paper, we propose a VQA method using motion-compensated temporal filtering (MCTF) and manifold feature similarity. To be more specific, a group of frames (GoF) is first decomposed into a temporal high-pass component (HPC) and a temporal low-pass component (LPC) by MCTF. Following this, manifold feature learning (MFL) and phase congruency (PC) are used to predict the quality of temporal LPC and temporal HPC respectively. The quality measures of the LPC and the HPC are then combined as GoF quality. A temporal pooling strategy is subsequently used to integrate GoF qualities into an overall video quality. The proposed VQA method appropriately processes temporal information in video by MCTF and temporal pooling strategy, and simulate human visual perception by MFL. Experiments on publicly available video quality database showed that in comparison with several state-of-the-art VQA methods, the proposed VQA method achieves better consistency with subjective video quality and can predict video quality more accurately.

## Introduction

The rapidly growing popularity of such digital consumer electronic devices as smartphones and portable computers has rendered video applications ubiquitous in our daily lives. Prior to being received by the users, video information needs to pass through several stages in communication systems, and is inevitably affected by noise and various kinds of distortion. Therefore, an accurate video quality assessment (VQA) method is needed to improve system performance and the quality of the users’ viewing experience.

Videos can be considered as orderly arrangements of several images, called frames. Therefore, a video contains both intra-frame spatial information and inter-frame temporal information. An effective VQA method should thus take both two aspects into consideration. In the last decade, the processing of spatial information in images has drawn an increasing amount of research interest. Due to a better understanding of the human visual system (HVS) [1–2] and advances in natural scene statistics (NSS) [3], a series of image quality assessment (IQA) methods have been proposed [4–5]. In the early stages of video quality evaluation, traditional IQA methods were used to predict the quality of each frame, and then computed the average of all frames’ qualities as an overall video quality [6]. The image quality of each frame clearly contributes considerably to overall video quality. However, such methods overlook the importance of temporal information, which limits their effectiveness. To overcome this disadvantage, several researchers have attempted to integrate temporal information into their methods. The relevant methods mainly used global motion to represent temporal information in video. Seshadrinathan *et al*. [7] considered motion information as a video feature and proposed motion-tuned, spatio-temporal quality assessment of natural video (MOVIE). To further investigate the HVS response to motion information in videos, Li *et al*. explored the effects of the spatio-temporal contrast sensitivity function. Meanwhile, by analyzing the characteristics of distortion in videos, a noise decoupling-based VQA method has been proposed [8]. Zhang et al. [9] exploited the visual masking effect to process the human perception of distortion in videos, and proposed a perception-based VQA method. In recent years, researchers have also attempted to simultaneously process spatial and temporal information by three-dimensional (3D) decomposition. In [10], Torkamani-Azar considered videos as 3D matrices, and used 3D singular value decomposition (3D-SVD) to extract 3D singular vectors as video features, and this 3D-SVD-based VQA method works well to evaluate video quality.

From the perspective of neuro-biology, the ultimate goal of VQA is to simulate the response of human visual systems. Previous studies have already revealed that manifold is fundamental to perception [11]. Given visual information like videos, which can be considered as a set of high-dimensional data, manifold learning aims at discovering the geometric properties existing inside the data. Therefore, it can be utilized to eliminate the redundancies in videos and extract the essence structure as video features. In recent years, several manifold learning methods have been proposed [12–14]. These methods have been widely used in several image/video processing fields, such as face recognition [15], image classification [16], etc. However, there is still less work focusing on applying manifold learning methods on predicting visual quality, especially for video quality.

According to above discussion, it is evident that the two most challenging issues in VQA are temporal description and simulation of human perception. Specifically, in this paper, we analyze wavelet coding theory [17] and consider its method of temporal decomposition to be a reference. We introduce motion-compensated view filtering (MCTF) from wavelet coding to decompose videos in the temporal domain. Then, in order to simulate human visual perception, Orthogonal Locality Preserving Projection (OLPP) algorithm [18] is employed to extract manifold feature. Finally, an asymmetric temporal pooling strategy is adopted to obtain an overall video quality. This newly proposed VQA method takes the following unique features:

1. According to the contracture of video, we utilize MCTF to decompose video into different frequency components;
2. By analyzing the characteristics of different frequency components, we deploy appropriate method to evaluate each component’s quality and integrate both qualities into video quality;
3. To ensure the VQA method incorporate with human visual characteristics, we use manifold learning as a perceptual approach to extract features.

The rest of this paper is organized as follows: Section 2 introduces each part of the proposed VQA method in detail. Experiments conducted on the Laboratory for Image & Video Engineering (LIVE) video quality database are described in Section 3. Directions for further research in the area are discussed in Section 4.

## Materials and methods

**Fig 1:**
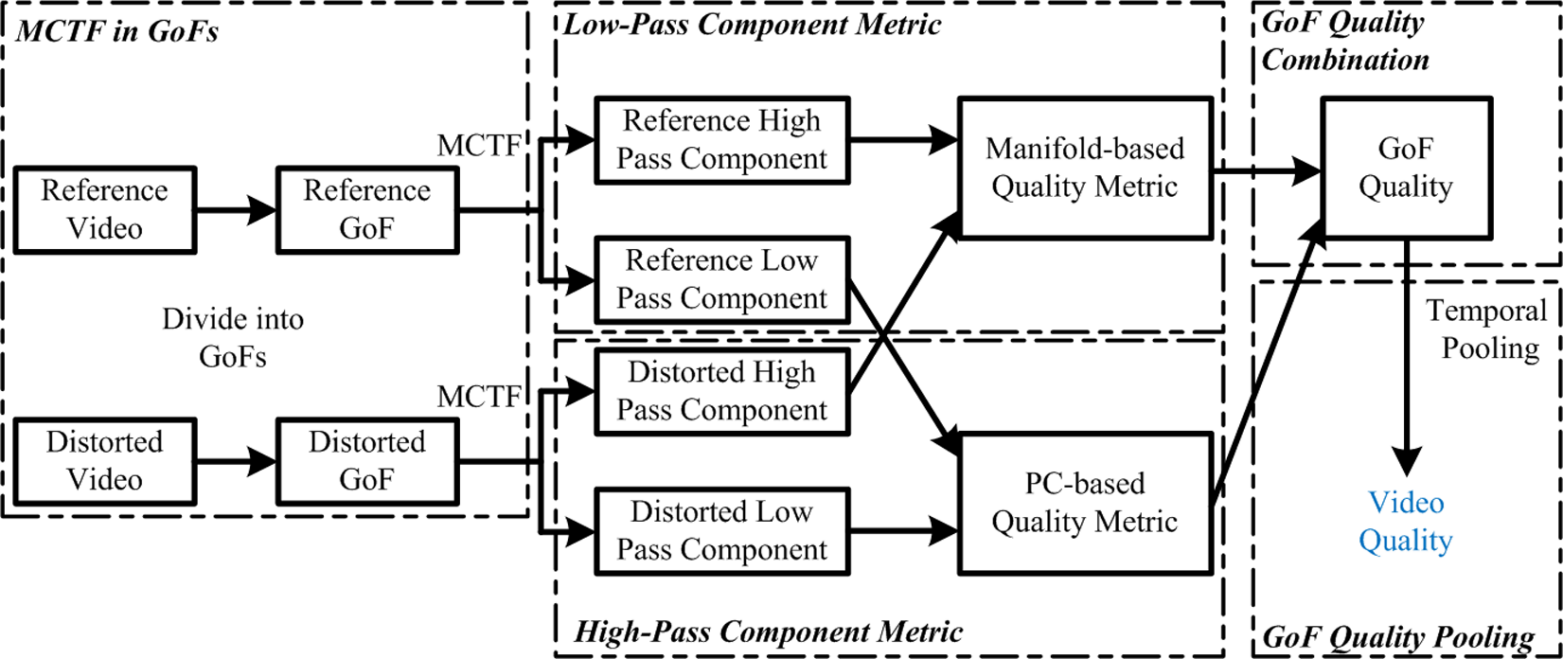
Framework for the proposed VQA method.

To address the difficulty in representing temporal information, we deal with it at both group of frame (GoF) level and video level. At the GoF level, MCTF is used to decompose temporal information into two different parts, namely, temporal high-pass component (HPC) and temporal low-pass component (LPC), whereas at the video level, a temporal pooling strategy is adopted. In order to accurately predict the qualities of both the HPC and the LPC, we use manifold learning and phase congruency (PC) similarity to simulate human visual perception. Based on this analysis, we propose a video quality assessment method using MCTF and manifold feature similarity. Fig 1 shows the framework of the proposed VQA method. It consists of five sequential processing modules. GoFs are first decomposed into a temporal HPC and a temporal LPC by MCTF. The quality of each temporal HPC and temporal LPC is then separately assessed, following which they are integrated as GoF quality. Finally, an overall video quality is obtained by the temporal pooling strategy of all GoF qualities.

### Temporal filtering in GoFs

It is well known that different frequency components consist varying information in an image, and distortion appears in different frequency components unevenly degrades image quality. This effect also exists in video. Specifically, the lower frequency component consists of still objects and structural information in the video, whereas the higher frequency component represents detail information concerning moving objects. As a result, it is necessary to decompose the video into different frequency components and appropriately predict the quality of each.

Traditional temporal filters directly decompose pixels at the same location in several frames. Because temporal motion widely exists in videos, which leads to scene displacement in adjacent frames, traditional temporal filters cannot thoroughly decompose video. In proposed VQA method, we use MCTF to implement temporal filtering in GoFs. The MCTF can decompose GoFs along the trajectory of the motion objects and archive better decomposition performance. In implementing MCTF on a GoF, two adjacent frames are filtered firstly. The filtering procedure can then be divided into two steps: motion compensation (MC) and temporal filtering (TF). In the MC step, let *l*_*n+1*_ and *l*_*n*_ denote two adjacent frames in a GoF. We first take *l*_*n*_ as a reference frame and search the matching block in *l*_*n+1*_ using a three-step search algorithm [19] to obtain motion vector *mv*_*n+1→n*_. Then, the mapping from *l*_*n*_ to *l*_*n*+1_, denoted by *M*_*n+1→n*_ can be acquired by using the motion vectors of both vertical and horizontal directions. Eventually, *M*_*n+1→n*_ can be subsequently used to transform *l*_*n*+1_ to motion-compensated frame *MC*_*n*+1_ using

$$MC_{n+1}[x+mv_{n+1\to n}^{H}(x,y),y+mv_{n+1\to n}^{V}(x,y)]=l_{n+1}(x,y)$$ (1)

where $mv_{n+1\to n}^{H}(x,y)$ is the horizontal motion vector from *l*_*n*+1_ to *l*_*n*_ in (*x*, *y*), and $mv_{n+1\to n}^{V}(x,y)$ is the horizontal motion vector from *l*_*n*+1_ to *l*_*n*_ in (*x*, *y*).

In the TF step, we use a lifting-based technique to decompose *l*_*n*_ and *MC*_*n*+1_. The lifting technique is an efficient implementation of the wavelet transform that uses low memory and is not computationally complex. Let *H* denote temporal HPC and *L* denote temporal LPC. The detailed implementation of decomposing *l*_*n*+1_ and *MC*_*n*+1_ can be represented by Eq (2):

$$\{\begin{array}{l} H(x,y)=\frac{1}{\sqrt{2}}[l_{n}(x,y)-MC_{n+1}(x,y)]\\ H(x,y)=\frac{1}{\sqrt{2}}\left\{l_{n}(x,y)+\frac{1}{2}\left[MC_{n+1}(x,y)-H(x,y)\right]\right\} \end{array}$$ (2)

where, (*x*, *y*) represents pixel location.

**Fig 2:**
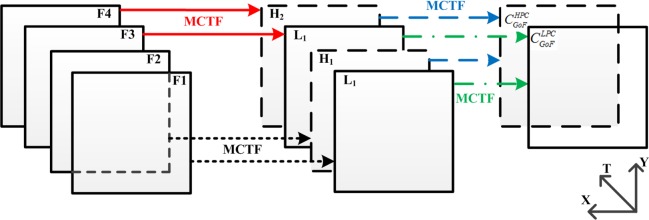
MCTF procedure in a four-frame GoF. Different levels of MCTF are separated by colors and line styles.

Fig 2 shows the implementation of MCTF in a four-frame GoF. Following the decomposition of adjacent frames (F1 and F2, F3 and F4), two temporal HPC frames (H1, H2) and two temporal LPC frames (L1, L2) are obtained. Let *CHPC GoF* and *CLPC GoF* denote the temporal HPC and the temporal LPC of the GoF, respectively. Then, *CHPC GoF* can be derived by MCTF in *H*_1_ and *H*_2_, and *CLPC GoF* is obtained by deploying MCTF to *L*_1_ and *L*_2_.

**Fig 3:**
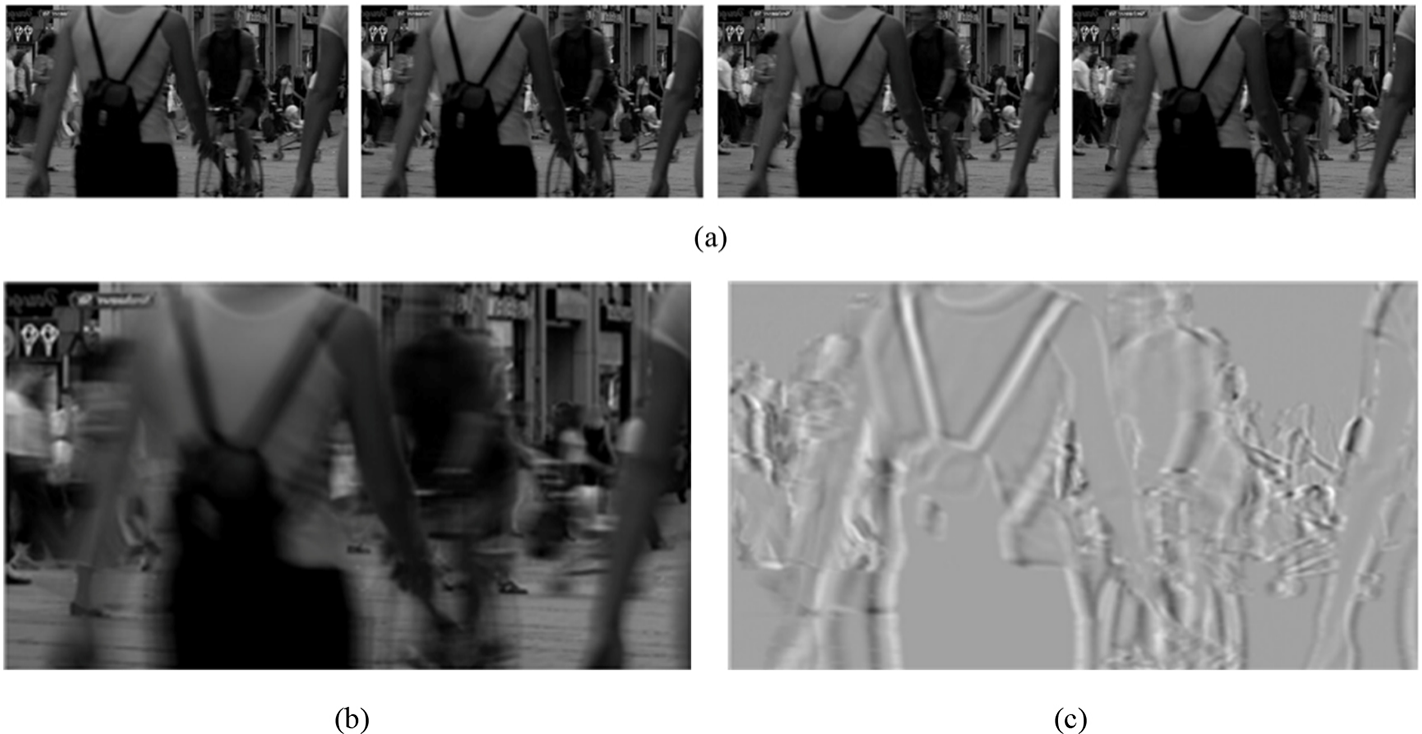
The original frames and the corresponding results of MCTF of the first GoF in video “Pedestrian Area” form the LIVE database. (a) 1^st^ GoF in “Pedestrian Area”. (b) Temporal Low-pass component. (c) Temporal High-pass component.

Note that distortion, such as blurring or blockiness, can change the result of block-based motion estimation, which affects the results of motion vector searching and causes the performance degradation of MCTF. Consequently, when a distorted GoF is processed by MCTF, we use the motion vector obtained from corresponding reference GoF instead. Fig 3 shows the result of implementing MCTF in a four-frame GoF, which is a randomly picked GoF in a video called “Pedestrian Area” from the LIVE video database. According to Fig 3(B) and 3(C), the temporal HPC consists of detail information regarding moving objects, whereas the temporal LPC contains the structural information of original scenes in the GoF and reserves all still objects.

### Temporal LPC quality metric

Natural scene videos are highly structured and can be seen as a high-dimensional set of data. Therefore, manifold learning can be applied in video to reduce the data’s dimension and extract the low-dimensional features, which can accurately reflect the intrinsic property of the video. From the results of the MCTF in Fig 3(B), we can find out that temporal LPC contains the most content and scene in the original GoF, and perceives to be quite similar to the original. Thus, it is reasonable to conclude that temporal LPC retains the most essential characteristics of original videos. Based on above analysis, we use manifold learning in the proposed VQA method to extract the distorted features of temporal LPC.

#### Feature extraction matrix learning

To extract the manifold feature from temporal LPC, firstly, 10,000 overlapped image patches with a size of 8×8 are randomly picked to build a training set. Next, the OLPP algorithm are employed to obtain the projection matrix in training set. Finally, the projection matrix, which is utilized as feature extraction matric, are used to extract the manifold features. The specific implementation of feature extraction matrix learning is stated as follows.

Before using OLLP to train the feature extraction matrix, we first apply principle component analysis (PCA) to reduce the dimension of input sample **Y** and only retain first 8 principle components of **Y** for training (the detail implementation of PCA can be referred in [20]). Meanwhile, [21] indicated that whitening can be used to simulate the working mechanism that Lateral Geniculate Nucleus (LGN) process visual information. Therefore, Y can be whitened into **Y**^**w**^ by

$$Y^{w}=Y\times W$$ (3)

where **W** is the whitened matrix, it can be calculated by eigenvalue and covariance matrix of **Y.**

Let *G* denote a graph with *m* nodes, and the *n*th node represents the whitened sample **y***w n*. Two nodes can be linked when they are adjacent, i.e., **y***w a* is among the nearest neighbors of **y***w b*. Moreover, if node *a* and node *b* are linked, the weight *S*_*ab*_ can be set as $e^{-{\left\|y_{a}^{w}-y_{b}^{w}\right\|}^{2}}$, otherwise *S*_*ab*_ = 0. In order to model the local manifold structure, we define *S* as weight matrix.

Then, the diagonal matrix **Φ** can be acquired by $\Phi_{aa}=\sum_{a=1}^{N}S_{ab}$, and the Laplacian matrix **L** is calculated as L = **Φ**—**S**. Let {**p**_1_, …, **p**_*n*_} denote the orthogonal basis vector, it can be defined as

$$\{\begin{array}{l} p_{n}=V_{\lambda_{\min}}\left[{\left({\bar{Y}}^{w}\Phi {\bar{Y}}^{wT}\right)}^{-1}{\bar{Y}}^{w}L{\bar{Y}}^{wT}\right],\;n=1\\ p_{n}=V_{\lambda_{\min}}\{\left\{I-{\left({\bar{Y}}^{w}\Phi {\bar{Y}}^{wT}\right)}^{-1}P^{\left(n-1\right)}{\left[Q^{\left(n-1\right)}\right]}^{-1}{\left[P^{\left(n-1\right)}\right]}^{T}\right\}\\ \;\cdot {\left(Y^{w}\Phi Y^{wT}\right)}^{-1}Y^{w}LY^{wT}\},\;\mathrm{others} \end{array}$$ (4)

where *V*_*λmin*_ represents the eigenvector corresponding to the smallest non-zero eigenvalue, and the orthogonal basis function, denoted by *F*_*Q*_, is expressed as

$$F_{Q}{}^{\left(n-1\right)}={\left[P^{\left(n-1\right)}\right]}^{T}{\left(Y^{w}\Phi Y^{wT}\right)}^{-1}P^{\left(n-1\right)}$$ (5)

Let M = {**p**_1_, …, **p**_*n*_} denote the transformation matrix. According to the PCA result, *r* is set to 8. Finally, the transformation matrix should be transformed from whitened space to original one as Eq (6) illustrated,

$$M_{opt}=W\times M$$ (6)

where **M**_*opt*_ is the optimal projection matrix, which can be used to extract image’s manifold feature.

**Fig 4:**
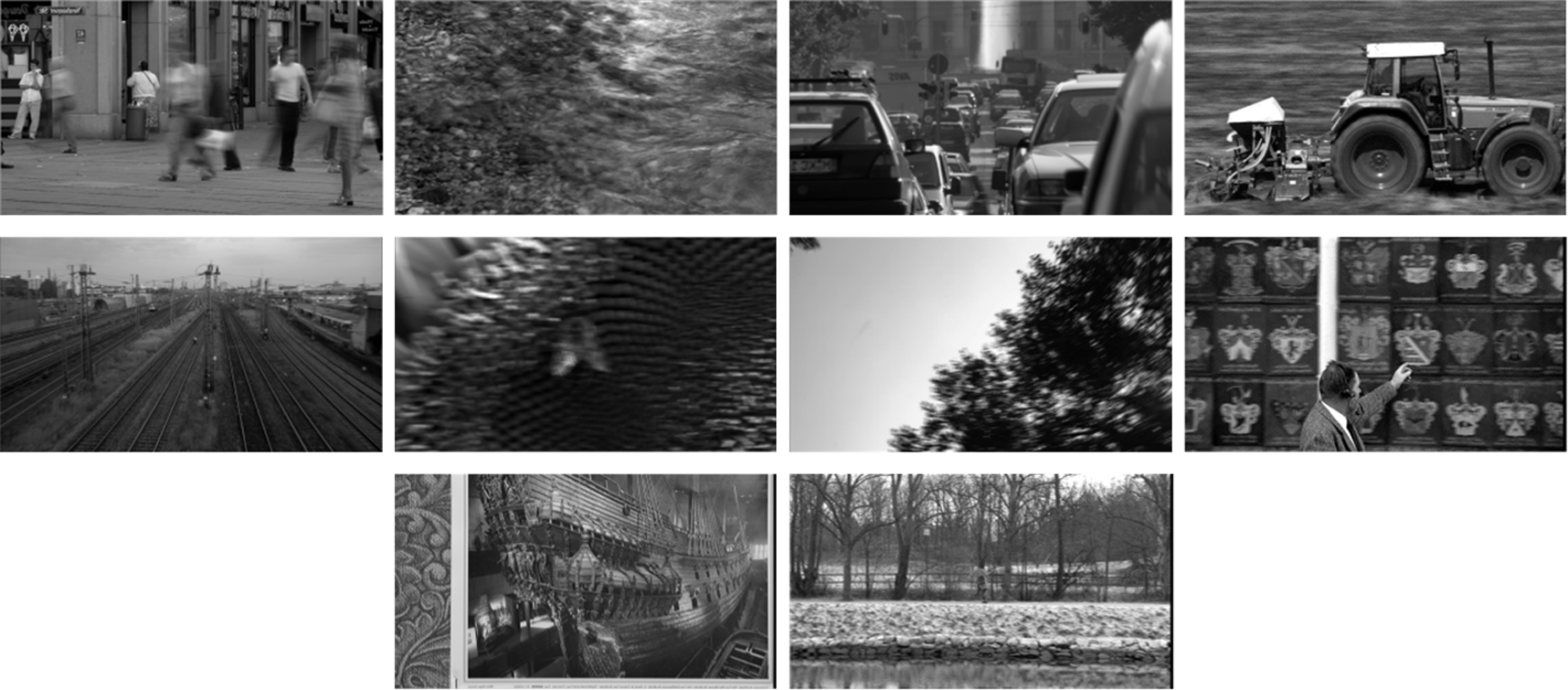
Training set from randomly selected temporal LPCs

It should be noted that in order for the optimal projection to more accurately reflect the essential features of temporal LPC, we used the temporal LPC to construct the training set. Specifically, we randomly selected 10 GoFs from reference videos in the LIVE video quality database and extracted 10,000 blocks from their temporal LPCs as the training set. Fig 4 shows the training set selected for the proposed VQA method.

#### Manifold feature similarity

The previously obtained 8×8 optimal projection matrix **M**_*opt*_ can then be used to extract the manifold features. Let *CLPC ref*(*i*) and *CLPC dis*(*i*) denote blocks in the reference and the distorted temporal LPC, and *MFref i* and *MFdis i* denote the manifold features of the reference and the distorted temporal LPC, which can be obtained by Eq (7) and Eq (8):

$$MF_{i}^{ref}(k_{1},k_{2})=\sum_{k_{1}=1}^{8}\sum_{k_{2}=1}^{8}C_{ref}^{LPC}\left(k_{1},k_{2}\right)\cdot M_{opt}\left(8-k_{1},8-k_{2}\right)$$ (7)

$$MF_{i}^{dis}(k_{1},k_{2})=\sum_{k_{1}=1}^{8}\sum_{k_{2}=1}^{8}C_{dis}^{LPC}\left(k_{1},k_{2}\right)\cdot M_{opt}\left(8-k_{1},8-k_{2}\right)$$ (8)

After obtaining the manifold features, the next step is to calculate the image block qualities by using these features. According to the similarity measurement defined in SSIM (Structural Similarity), the manifold feature similarity for each block in temporal LPC can be acquired as Eq (9). This is a commonly used method to calculate the similarity between two positive number sets. The result of Eq (9) is in the range of 0 to 1, and 1 implies a perfect match between two set of numbers.

$$qblock_{i}^{LPC}=\frac{2\times MF_{ref}(i)\times MF_{dis}(i)+C_{1}}{MF_{ref}{}^{2}(i)+MF_{dis}{}^{2}(i)+C_{1}}$$ (9)

where *C*_1_ is a small constant to ensure that the denominator is non-zero,

Then, the quality of temporal LPC can be integrated by averaging all blocks’ qualities. Let *q*^*LPC*^ denote the quality of temporal LPC. It can be obtained as

$$q^{LPC}=\frac{1}{k}\sum_{i=1}^{k}qblock_{i}^{LPC}$$ (10)

where *k* is the total number of blocks in an LPC.

### Temporal HPC quality metric

As previously mentioned in Section 2, the temporal HPC of a GoF contains information regarding moving objects and related details. Phase is an important image feature that captures a considerable amount of detail information of an image. As a result, phase feature can be used to evaluate the quality of temporal HPC. Previous researches have revealed that the HVS is highly sensitive to pixels with high phase congruency (PC) [22]. Thus, in the proposed VQA method, we extract the PC from temporal HPC as distortion features

It is well-known that the visual cortex can be satisfactorily simulated by the Log-Gabor filter. In this paper, we use the responses of the Log-Gabor filter to calculate the PC of the temporal HPC. Specifically, Eq (11) is adopted to calculate PC. The detail explanation of Eq (11) can be found in [22].

$$PC(m)=\frac{E_{o}(m)}{\varepsilon +\sum_{S}A_{s,o}(m)}$$ (11)

where *A*_*s*,*o*_(*m*) represents local amplitude of response in Log-gabor filter, and *E*_*o*_(*m*) denotes local energy. *s* and *o* is defined as scale and orientation of the filter. *ε* is a small constant to avoid denominator being zero.

Following the calculation of the PC features as above, we predict the quality of the temporal HPC using these features. Let *PCHPC ref* and *PCHPC dis* denote the PC features of the reference temporal HPC and the distorted temporal HPC, respectively. The similarity measurement method used in manifold feature similarity calculation is then used to obtain the quality of the distorted temporal HPC, as illustrated in Eq (12):

$$q_{HPC}=\frac{2\times PC_{ref}^{HPC}\times PC_{dis}^{HPC}+C_{2}}{{\left(PC_{ref}^{HPC}\right)}^{2}+{\left(PC_{dis}^{HPC}\right)}^{2}+C_{2}}$$ (12)

where *C*_2_ is a small positive constant to ensure a non-zero denominator.

### GoF quality pooling

The quality of *n*th GoF, denoted by *qGoF n*, can be obtained by a combination of the quality of the temporal HPC *qHPC n* and that of the temporal LPC *qLPC n*. In the proposed VQA method, we use a linear weighting summation model to calculate *qGoF n* as Eq (13) illustrated. Because video processing contains huge data volume, we adopt linear summation instead of more sophisticated regression model for its low computational complexity.

$$q_{n}^{GoF}=\omega_{1}\cdot q_{n}^{HPC}+\omega_{2}\cdot q_{n}^{LPC}$$ (13)

where *ω*_1_ and *ω*_2_ are the weights assigned to temporal HPC and temporal LPC. It is expected that the quality of temporal LPC has lager impact on overall GoF quality and so *ω*_1_ <*ω*_2_. In our method, *ω*_1_ is set to 0.3 through performance tuning, and *ω*_2_ is set to 0.7. Details reason of these determinations is illustrated in Section 3.2.

### Temporal pooling

Following the calculation of the qualities of all GoFs, a VQA method needs to integrate all GoF qualities into an overall video quality. However, simply averaging all GoF qualities does not consist with human perception, and is likely to degrade the performance of prediction. It is thus necessary to simulate several HVS characteristic while combining GoF qualities into video quality. Because observers are more sensitive to degradation in video quality than improvement, we adopt the implementation presented in [23] to simulate such asymmetric responses to fluctuation in GoF qualities. We first adjust GoF quality *qGoF n* to intermediate GoF quality *qGoF' n* according to Eq (14):

$$q_{n}^{GoF}{}^{'}=\{\begin{array}{c} q_{n-1}^{GoF}{}^{'}+a^{+}q_{\Delta }^{GoF},\;q_{\Delta n}^{GoF}>0\\ q_{n-1}^{GoF}{}^{'}+a^{-}q_{\Delta }^{GoF},\;q_{\Delta n}^{GoF}\leq 0 \end{array}$$ (14)

where *a*^+^ and *a*^-^ embody asymmetric behavior, and

$$q_{\Delta n}^{GoF}=q_{n}^{GoF}{}^{'}-q_{n-1}^{GoF}$$ (15)

In the proposed VQA method, *a*^+^ is set to 0.09 and *a*^-^ to 0.8 through performance tuning.

Finally, the overall video quality *Q* is calculated by averaging all the intermediate GoF quality.

$$Q=\frac{1}{N}\sum_{n=1}^{N}q_{n}^{GoF}{}^{'}$$ (16)

where *N* is the number of GoFs in a video.

## Results and discussion

### Subjective database and performance index

The LIVE video quality [24] database is used to evaluate the performance of the proposed VQA method. The LIVE video quality database consists of 10 reference videos and 150 distorted videos generated from the reference videos. The distorted videos are created by using four types of distortion: Wireless network transmission Distortion (WD), IP network transmission (IP) distortion (IP), H.264 compression distortion (H264), and MPEG-2 compression distortion (MPEG-2). All reference videos and distorted videos have a resolution of 768×432 and the frame rate ranged from 25 fps to 50 fps.

Three measures are employed as performance indexes to evaluate the performance of the proposed VQA method: the Pearson linear correlation coefficient (PLCC), Spearman’s rank-order correlation coefficient (SROCC), and root-mean-square error (RMSE). Detailed formulation regarding these can be reviewed in [25]. In general, a higher PLCC represents better correlation between predicted quality and subjective assessment of quality. The SROCC measures the monotonicity of predicted quality, whereas the RMSE measures error in predicted quality. A smaller RMSE indicates better prediction performance.

### Parameterization

**Fig 5:**
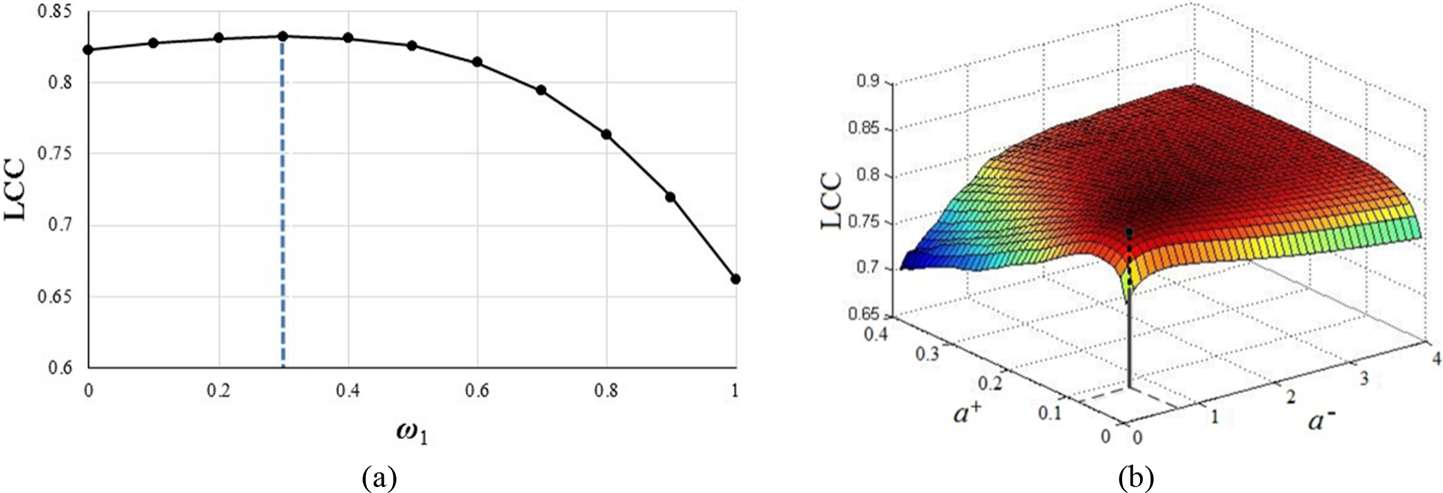
Parameter determinations in the proposed VQA method. (a) Tuning Performance of *ω*_1_. (b) Tuning Performance of *a*^+^ and *a*^-^.

Two sets of parameters need to be determined in the proposed VQA method, *i*.*e*., *ω*_1_, *ω*_2_ and *a*^+^, *a*^-^. In tuning *ω*_1_ and *ω*_2_, both *a*^+^ and *a*^-^ are set to 1. *ω*_1_ is changed from 0 to 1 in increments of 0.1. Fig 5(A) shows the result of tuning of *ω*_1_. When *ω*_1_ is set to 0.3, the highest value of the PLCC is obtained. Since *ω*_1_ + *ω*_2_ = 1, *ω*_2_ is set to 0.7. The tuning result shows that the quality of the temporal LPC contributes most to overall quality. It further confirms that the temporal LPC contained the greatest number of items of information from the original video. Once *ω*_1_ and *ω*_2_ are fixed, *a*^+^ and *a*^-^ are set by the same method. *a*^+^ is changed from 0 to 0.4 in increments of 0.01, and *a*^-^ is changed from 0 to 4 in increments of 0.1. The results can be seen in Fig 5(B). When *a*^+^ = 0.09, *a*^-^ = 0.8, the PLCC reaches its peak. The significant difference in value confirma the hypothesis of the asymmetry of human responses to fluctuations in quality: to wit, observers are more sensitive to quality degradation than quality improvement while watching videos.

### Determination of GoF size

In proposed method, the MCTF is implemented on GoFs. Therefore, it is necessary to explore whether the GoF size will have effect on final performance. As the requirement in implementation of lifting-based wavelet transform, the GoF number should be set as *n*-th power of 2. Consequently, Table 1 is listed to illustrate the effect of GoF size.

**Table 1:** Performance indicators of proposed VQA method with different GoF numbers.

GoF number	PLCC	SROCC	RMSE
**4**	0.8472	0.8298	4.9764
**8**	0.8471	0.8300	4.9788
**16**	0.8464	0.8271	4.9889
**32**	0.8217	0.8003	5.1324
**64**	0.8035	0.7961	5.4231

By observing the experimental results in Table 2, it can be conclude that the performances are nearly equivalent while the GoF numbers are 4, 8, or 16, and when the GoF number is greater than 32, the performance degraded sharply. Furthermore, in implementation, the larger GoF number will inevitably introduce tremendous computational complexity. Therefore, in proposed VQA method, the final GoF number is set as 4.

**Table 2:** PLCC comparison for each module of the proposed VQA method.

	Details	WD	IP	H264	MPEG	ALL
Plan-A	MC+TP	0.8406	0.7711	0.7283	0.8384	0.8353
Plan-B	PTS+TP	0.8255	0.7671	0.7462	0.8136	0.8107
Plan-C	MC+PTS	0.8280	**0.7913**	**0.7506**	0.8247	0.8235
Proposed method	MC+PTS+TP	**0.8591**	0.7773	0.7385	**0.8570**	**0.8472**

MC: Motion compensation in temporal filtering. PTS: Proper training set (randomly selected temporal LPC) in MFL. TP: using temporal pooling strategy to combine GoF quality measures.

### Impact of each module in the proposed VQA method

To verify the impact of each module employed in the proposed VQA method, we design another three plans that partly choose several modules in proposed method for comparison, denoted by Plan-A, Plan-B and Plan-C, respectively. In Plan-A, we use natural image instead of randomly picked temporal LPC to build training set, and other implementations are same with proposed method. In Plan-B, motion compensation is not included in temporal decomposition, and other implementations are same with the proposed method. In Plan-C, all GoF qualities are averaged to yield the overall video quality and other implementation are same with proposed method. By comparing Plan-A with proposed method, we can conclude that the optimal projection matrix trained by a proper training set coincides to a greater extent with human perception. The performance improvement between Plan 2 and the proposed method shows that motion compensation profits the temporal filtering in the temporal decomposition of GoFs. Finally, by using the temporal pooling strategy instead of simply averaging the quality measures of all GoFs, the proposed method outperforms Plan 3. In summary, each module employed in the proposed VQA method plays a positive role in performance improvement.

### Overall prediction performance

Table 3 lists the performance indexes of the proposed VQA method in the LIVE video quality database. For comparison, we also provide the results of several methods in Table 4. These comparative methods include the traditional image quality metric with temporal averaging (PSNR, SSIM), methods using motion information (MOVIE, the method proposed in [8]), method simulating the HVS working mechanism (the metric proposed in [9]), and method adopting 3D transformation (VRF) and the method standardized by Video quality expert group (VQEG) [26]. The best performance is shown in bold in the table. We see from Table 4 that the proposed methods achieve the best indexes for distortion like WD, IP and MPEG-2. As for H264 distortion, the indexes of proposed VQA method are not as accurate as other VQA methods. Essentially, the H.264 compression will introduce blocking effect in videos. Meanwhile, PC feature is much more sensitive to those artificial edges caused by blocking effect than human eyes. Therefore, slight quality degradation caused by block effect can be exaggerated by PC-based quality metric adopted in proposed method. As a result, the prediction accuracy of proposed method will be declined for H.264 compressed video. However, the proposed VQA method outperformed all other VQA methods in terms of overall performance for all distorted videos (ALL) in the LIVE video quality database. Taking all indicators into consideration, the proposed VQA methods yield the highest correlation with subjective quality and can predict video quality more accurately.

**Table 3:** Performance on separate types of distortion.

	PLCC	SROCC	OR	RMSE
WD	0.8591	0.8359	0	4.8332
IP	0.7573	0.7513	0	6.4879
H264	0.7385	0.7230	0.025	7.6598
MPEG-2	0.8570	0.8414	0	4.9124
ALL	0.8472	0.8298	0	4.9764

**Table 4:** Performance comparison on the LIVE VQA database.

	WD	IP	H264	MPEG2	ALL
PSNR	0.4675	0.4108	0.4385	0.3856	0.4035
SSIM	0.5401	0.5119	0.6656	0.5491	0.5444
VQM	0.7325	0.648	0.6459	0.786	0.7236
MOVIE	0.8386	0.6772	0.7902	0.7595	0.8116
STAQ	0.5684	0.7080	**0.8778**	0.7988	0.7192
VRF	0.7708	0.7453	0.7062	0.6019	0.6983
Metric in [8]	—	—	—	—	0.8460
Metric in [9]	0.8280	0.7010	0.8160	0.7420	0.8150
Proposed*	**0.8591**	**0.7573**	0.7385	**0.8570**	**0.8472**

**Fig 6:**
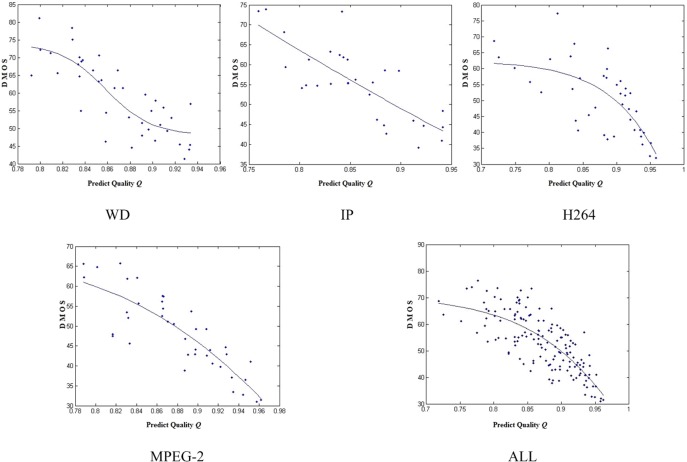
Scatter plots of proposed VQA metric. All distortion types in LIVE video databases are listed.

We also show the scatter plots of the proposed VQA method in Fig 6. The horizontal axis denotes the predicted qualities obtained by proposed method and the vertical axis denotes the subjective qualities provided by LIVE database. These scatter plots reflect the approximate linear correlation between the prediction qualities and subjective qualities. Fig 6 shows that the predictive quality of the proposed VQA method was highly correlated with subjective assessments of quality.

## Conclusions

In this paper, we propose a video quality metric using motion-compensated temporal filtering (MCTF) and manifold feature similarity. The main idea underlying this method is to decompose videos in the temporal domain and appropriately predict the qualities of temporal LPC and temporal HPC generated from temporal decomposition. Specifically, we use MCTF to decompose a GoF into different frequency components. According to the characteristics of both the frequency components and human perception characteristics, we extract manifold features and the phase congruency in temporal LPC and temporal HPC, respectively, and then calculated feature similarity as GoF quality. Finally, a temporal pooling strategy is used to obtain an overall video quality. Experiments on the LIVE video quality database shows that the proposed VQA method performed satisfactorily in predicting video quality. In future work, some outstanding issues need to be considered, such as a better temporal pooling strategy as well as a temporal decomposition method.
